# Extraordinary transmission of gigahertz surface acoustic waves

**DOI:** 10.1038/srep33380

**Published:** 2016-09-19

**Authors:** Sylvain Mezil, Kazuki Chonan, Paul H. Otsuka, Motonobu Tomoda, Osamu Matsuda, Sam H. Lee, Oliver B. Wright

**Affiliations:** 1 Division of Applied Physics, Graduate School of Engineering, Hokkaido University, Sapporo 060-8628, Japan; 2 Institute of Physics and Applied Physics, Yonsei University, Seoul 120-749, Korea

## Abstract

Extraordinary transmission of waves, i.e. a transmission superior to the amount predicted by geometrical considerations of the aperture alone, has to date only been studied in the bulk. Here we present a new class of extraordinary transmission for waves confined in two dimensions to a flat surface. By means of acoustic numerical simulations in the gigahertz range, corresponding to acoustic wavelengths *λ* ~ 3–50 μm, we track the transmission of plane surface acoustic wave fronts between two silicon blocks joined by a deeply subwavelength bridge of variable length with or without an attached cavity. Several resonant modes of the structure, both one- and two-dimensional in nature, lead to extraordinary acoustic transmission, in this case with transmission efficiencies, i.e. intensity enhancements, up to ~23 and ~8 in the two respective cases. We show how the cavity shape and bridge size influence the extraordinary transmission efficiency. Applications include new metamaterials and subwavelength imaging.

The subject of extraordinary optical transmission through an array of subwavelength holes arose from measurements in the far infra-red and visible wavelength ranges in metal apertures^1^,^2^. This work inspired extensive studies on the analogous extraordinary acoustic transmission phenomenon. This was theoretically predicted for bulk waves^3^,^4^,^5^,^6^,^7^,^8^,^9^ and experimentally verified in a wide variety of grating, slit and hole systems^10^,^11^,^12^,^13^,^14^,^15^,^16^,^17^,^18^,^19^. Several types of transmission mechanism were proposed for acoustic extraordinary transmission, in particular periodic-lattice resonances, Fabry-Perot-type resonances, elastic Lamb-mode-resonances, Helmholtz resonators, membrane resonances and space coiling. Experiments on the passage of Rayleigh waves through a fluid channel have demonstrated anomalously low acoustic transmission at certain frequencies^20^. However, in spite of the interesting possibilities in the fields of metamaterials and subwavelength imaging, the extraordinary transmission of surface waves, and in particular surface acoustic waves, has never been investigated. This is surprising in view of the potential simplifications introduced by reducing the dimensionality of the extraordinary transmission problem to waves confined to a plane, with potential applications in miniaturization of the overall geometry.

In this paper we demonstrate by means of numerical simulations the phenomenon of extraordinary transmission of surface acoustic waves in solids. We first consider the case of a straight waveguide in the form of a deeply subwavelength-width bridge joining two blocks. We also consider a bridge structure containing a resonant cavity. With these structures we demonstrate transmission efficiencies up to ~23, calculated from the intensity enhancement over a region sampling the transmitted surface acoustic field. For both types of structure we choose microscopic sizes in order to give acoustic resonances in the gigahertz range, as such frequencies correspond to those used in surface acoustic wave filters and devices. Furthermore, direct surface acoustic wave imaging techniques exist for this frequency range^21^,^22^,^23^,^24^, and so our work is therefore experimentally realizable.

## Simulations

**Figure 1:**
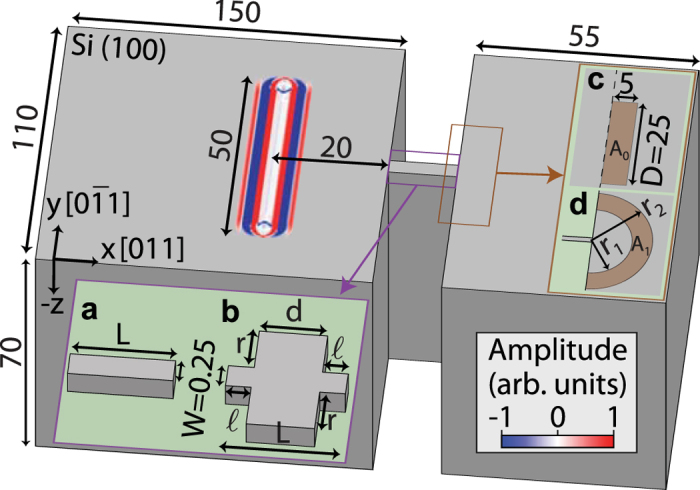
Schematic representation of the simulated sample with a deeply-subwavelength-width surface-acoustic waveguide in the form of a bridge joining two Si (100) blocks. Surface acoustic waves are generated with a line source of length 50 μm at a distance of 20 μm from the sample edge. All dimensions are in microns. Insets (**a**,**b**) depict the top part of the bridge of length *L* without (**a**) and with (**b**) a cavity resonator of cross section (2*r* + *W*) × *d*, where *W* = 0.25 μm is the minimum bridge width and the length 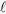 pictured is the length of the straight bridge sections. Insets (**c**,**d**) respectively depict the rectangular and annular areas (*A*_0_ and *A*_1_, respectively) used to determine the transmission efficiency. The colour scale refers to the simulated surface wave normal particle velocity corresponding to the wave field calculated 0.4 ns after excitation.

The sample consists of a crystalline Si (100) substrate divided into three regions— two blocks and a connecting bridge— as shown in Fig. 1. Silicon was chosen because of the relative ease of future sample preparation. The left-hand (right-hand) block is of dimensions 150 × 110 μm^2^ (55 × 110 μm^2^) as seen from the top. The bridge connecting the two blocks is of lateral thickness *W* = 0.25 μm and variable length *L* (as shown in inset (a)), the former dimension chosen to be much smaller than the acoustic wavelength *λ* (~5 μm at 1 GHz, so *W* ~ *λ*/20 at this frequency). The bridge can contain a cavity in the form of symmetrical rectangular extensions of cross section *d* × *r* on both sides, where 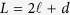 (see inset (b)), 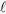 being the length of the straight bridge sections. The depth of the whole sample is chosen to be 70 μm, used with absorbing boundary conditions on the bottom surface and side surfaces except those involving the gap, bridge, and resonators, in order to mimic an infinite substrate. This geometry is similar to that used in previous experimental and numerical investigations in the context of phononic crystal waveguides^22^.

The acoustic source is chosen to correspond to that produced by a laser line source with a thermoelastic generation mechanism; we use a simplified elastic-dipole model^25^ with a spatial distribution of horizontal surface forces along the *x* axis (see Fig. 1) of magnitude proportional to 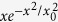 with *x*_0_ = 1 μm (where *x* = 0 corresponds to the centre of the source), applied as a line-source of length 50 μm along the *y*-axis. The temporal variation of the excitation is a step-like function (a quarter period of a sinusoid) with a 1 ns rise time. This acoustic source produces a spectrum of surface acoustic waves up to ~2 GHz as in experiments with sub-picosecond optical pulse excitation^21^,^22^,^23^,^24^. This acoustic source is applied to the left-hand block, as shown in Fig. 1, and the waves with components propagating in the *x* direction are monitored. For our choice of crystal cut (see Fig. 1), similar to that used in previous GHz surface acoustic wave imaging experiments on crystalline silicon^24^, the surface waves propagating at angles |*θ*| ≲ 22.4° to the *x* direction have largely pseudo-surface wave character^26^,^27^. As in optical interferometric detection experiments, two-dimensional data sets representing the out-of-plane (i.e. normal) particle velocity are recorded. Further details of the simulations are given in the Methods section.

## Results

### Straight bridge

**Figure 2:**
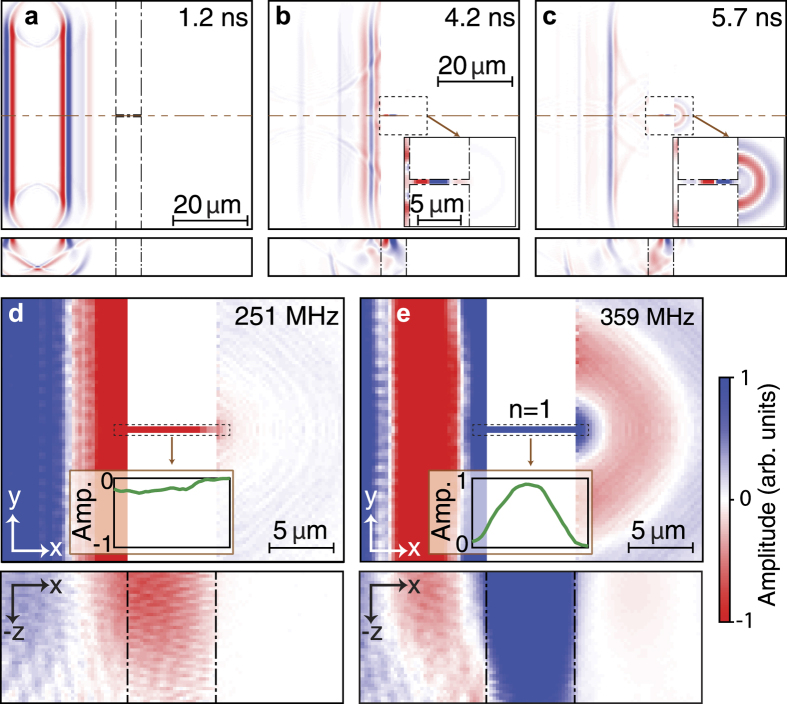
Simulations in the time and frequency domains of surface acoustic wave fields on transmission through a deeply-subwavelength-width bridge. (**a–c**) Simulated amplitude of the acoustic wave propagation in a subwavelength bridge of length *L* = 7 μm and width *W* = 0.25 μm, 1.2 ns (**a**) 4.2 ns (**b**) and 5.7 ns (**c**) after the acoustic wave generation, shown at the surface (*x*, *y*) (upper plots) and for vertical (*x*, *z*) sections (lower plots). (**a**) The vertical dotted-dashed lines represent the block boundaries. The insets in (**b**,**c**) depict close-ups near the bridge region. (**d**,**e**) show the amplitudes of the temporal Fourier transform respectively off resonance at 251 MHz (*η* = 0.5) and on resonance at 359 MHz (*η* = 5.7) at the surface (*x*, *y*) (upper plots) and for vertical (*x*, *z*) sections (lower plots). (**d**,**e**) include line-plot snapshots of the average out-of-plane particle velocity plotted along the bridge-length direction (*x*). The colour-scale bar is the same for (**a**–**c**) and (**d**–**e**), but with a sensitivity 4 times larger for the bottom images of (**d,e**) compared to the top ones. (The amplitude in some places in the upper plots of (**d**,**e**) saturates the colour scale up to a factor of ~10).

**Figure 3:**
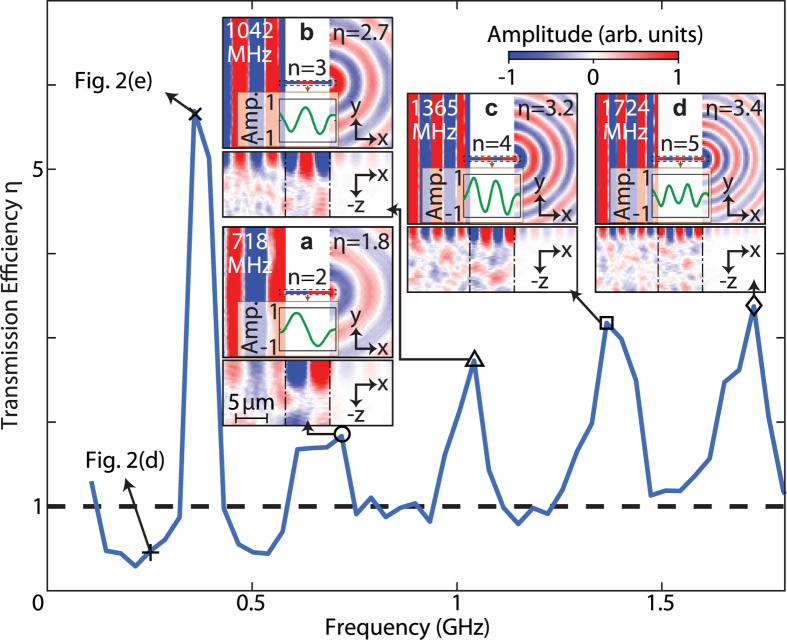
Simulated spectrum of the transmission efficiency *η*(*f*) for the straight bridge. The bridge length is *L* = 7 μm and the width is *W* = 0.25 μm. The condition *η* = 1 is indicated by the dashed line. Insets (**a**–**d**): amplitude of the temporal Fourier transform corresponding to four local maxima in *η* between 0.5 and 1.8 GHz at the surface (upper plots) and in (*x*, *z*) section (lower plots), including line-plot snapshots of the average out-of-plane particle velocity plotted along the bridge-length direction (*x*). The colour scale bar shown is the same for (**a**–**d**) and the same as in Fig. 2(d,e), with a scale 4 times more sensitive for the bottom images compared to the top ones.

An example of the simulated acoustic wave propagation in the time domain for the case of a straight bridge (as in inset (a) of Fig. 1) of length *L* = 7 μm aligned along the *x* direction is shown in Fig. 2(a–c). Acoustic amplitude transmission into the subwavelength bridge is clearly visible at *t* = 4.2 and at *t* = 5.7 ns (Fig. 2(b,c)), as is acoustic reflection from the edge parallel to the *y* direction. One can also notice the amplitude decrease of the reflected wave in Fig. 2(b,c) compared to the incident one in Fig. 2a, which is as expected since a part of the amplitude of the surface acoustic waves will propagate in the depth (−*z*-direction) at the block edge (see Methods). At 5.7 ns (Fig. 2c) one can easily discern transmitted waves after the bridge. Also shown in Fig. 2(a–c) by the lower plots are the depth profiles along the *x* direction at the same times. At short times some acoustic energy is radiated to bulk waves, whereas for longer times one can make out that guided surface waves persist after the bridge. These guided waves are discussed below in greater detail in the frequency domain. Owing to the excitation of bridge vibrational modes, the acoustic amplitude transmission to the right-hand block is expected to show a strong resonant frequency response characteristic of extraordinary acoustic transmission. To access the frequency domain, a temporal Fourier transform of the acoustic field is thus performed. The results are shown in Fig. 2(d,e) and in the insets (a–d) of Fig. 3: at 251 MHz, for example, there is very little visible transmission (Fig. 2(d)), whereas at 359 MHz there is evident transmission (Fig. 2(e)). Transmission is also evident in the insets (a–d) of Fig. 3 at selected frequencies up to 1724 MHz.

In order to obtain a quantitative measure of the transmission efficiency and to extract the frequencies associated with extraordinary acoustic transmission (EAT), we exploit the production of relatively simple wave fronts of the acoustic field in the absence of the subwavelength bridge (i.e. approximately plane wave fronts for *x*-directed propagation) and in its presence (i.e. approximately semicircular wave fronts centred at the output end of the bridge). In the absence of any bridge structure, acoustic wave fronts remain closely perpendicular to the *x* axis provided that acoustic diffraction is negligible for surface-wave propagation from the line source— a valid assumption in our case for distances well within the Rayleigh length *L*_*R*_ for diffraction (i.e., *L*_*R*_ = *πD*′^2^/*λ* ~ 800 μm, with *D*′ = 50 μm the length of the laser line source and *λ* ~ 10 μm a typical acoustic wavelength). One can therefore sample the Fourier modulus of the incident surface waves by integrating over a thin rectangular reference region (i.e. a rectangle of high aspect ratio) chosen perpendicular to the *x* direction in the absence of a bridge structure, as shown by the inset (c) of Fig. 1. This region, of dimensions 5 × 25 μm^2^, is chosen at a distance of *L* + 20 μm from the source, making use in the simulation of a seamless connection of the Si blocks. The sampled wave amplitude is essentially independent of the distance from the source in the region of the chosen rectangle position (i.e. well within the Rayleigh length from the source as defined above).

When the bridge is present, the transmitted wave fronts emanating from the subwavelength-width bridge exit are approximately circular in shape on Si (100)^26^,^27^,^28^, as is evident in Fig. 2(c–e) and in the insets of Fig. 3. (The surface waves propagating at angles |θ| ≲ 22.4° and for 67.6° ≲ |θ| ≲ 90° to the *x* direction have largely pseudo-Rayleigh-wave character, whereas those propagating at angles 22.4° ≲ |*θ*| ≲  67.6° to the *x* direction have largely Rayleigh-wave character^26^). In order to monitor the amplitude of the transmitted surface waves for comparison with the incident waves, it therefore suffices to integrate the Fourier modulus over a thin 180° annular region centred on the output end of the bridge (see Fig. 1(d)) of the same arc length, width and area as the thin rectangular reference area considered above. This region is chosen so that the arc is perpendicular to the surface-wave propagation direction after transmission through the bridge— a valid approach because for a single acoustic mode (as verified below in our geometry) for a given propagation direction, a measure of the local acoustic energy flow can be accurately obtained by sampling the surface acoustic field along a line perpendicular to the acoustic propagation direction, provided that the radius of the arc is large enough (i.e. ≳*λ*) for the effect of its curvature to be negligible. The thin semicircular arc in our case can initially be tentatively chosen with average radius 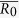 = 7.96 μm, corresponding to internal and external radii *r*_1_ = 5.46 and *r*_2_ = 10.46 μm, respectively, giving a mean arc length (*π*[*r*_1_ + *r*_2_]/2) equal to *D* = 25 μm and an annular thickness of 5 μm equal to that of the thin rectangle (see Fig. 1(c)), so that both regions have identical areas *A*_*0*_ = 125 μm^2^. This choice is, however, only suitable for frequencies ≳2 GHz, when *λ* ≲ 2.5 μm. For our range of experimental frequencies we therefore choose a larger mean radius 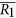 = 50 μm, with an annular thickness decreased by a factor 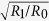 = 2.5 to 2 μm, i.e. corresponding to an area increased to *A*_1_ = 2.5*A*_0_, to account for the combined effects of the decrease in wave amplitude on propagation outwards (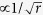 for radii *r* ≳ *λ*) and the increase in arc length (∝*r*); for a monochromatic circularly propagating wave in two dimensions, the radial form of the waveform contains a spatial modulation in the form *J*_0_(*kr*) (see, e.g., ref. 29), where *k* is the wavenumber, which for *kr *> 1/4 (i.e. *r *> *λ*/25) can be approximated as 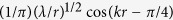. Larger values of the choice of *R*_1_ would lead to more accurate sampling in general, in particular at *f*~100 MHz, but the value *R*_1_ = 50 μm was the largest possible for the considered size of the simulated sample, limited by the computer memory. We estimate a maximum error ~20% on the values of *η* derived at *f* ~ 100 MHz.

The acoustic amplitude is thus sampled for incident and transmitted waves from the area-integrated modulus of the temporal Fourier transform of the acoustic field over areas *A*_0_ and *A*_1_ with and without the subwavelength bridge, respectively. An experimentally-accessible transmittance *T*(*f*) as a function of frequency *f* can thus be calculated from the ratio of these two area integrals: 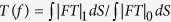 (where 0 and 1 refer to the reference and bridge-structure cases, respectively, and |*FT*| indicates the modulus of the temporal Fourier transform). In the absence of ultrasonic attenuation, *T* = 1 corresponds to the case of no energy loss, i.e. to perfect transmission.

A problem would arise with this definition if the acoustic field consisted of multiple modes that influence the surface field, as for example in the region of the source in Fig. 2(a) where bulk modes are also present. However, for our chosen sampling regions, this is not the case. We verified this in two ways. Firstly, one can see by examining the *x*-z vertical sections of the acoustic field to the right the bridge at different frequencies, as for the examples of Figs 2 and 3, that the only significant acoustic field is localized within the acoustic wavelength (*λ*) of the surface, as expected for a single surface mode. Secondly, we verified, as mentioned above, that the acoustic amplitude in the region of the sampling annulus *R*_1_ at each sampled frequency decays accurately as 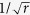, where *r* is the radial distance, again as expected for a single radially propagating surface mode for any specific propagation direction for radial distances *r *≳ *λ*.

In the presence of a bridge structure, one expects a transmission efficiency that depends on the amplitude transmittance *T*(*f*) and, in the limit of geometrical acoustics, to the ratio between *D* and *W*, where *D* is the length of the rectangular area *A*. A natural definition of the transmission efficiency *η* for acoustic intensity, analogous to the optical case^2^, is therefore


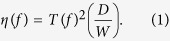


For wavelengths 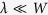 one expects from geometrical acoustics in the absence of material losses that *η* = 1. (Intrinsic ultrasonic attenuation in crystalline Si over propagation distances ~100 μm are negligible at the frequencies in question in this paper^30^). The condition *η*(*f*) > 1 for a particular frequency *f* corresponds to a transmission that is greater than expected, i.e. to EAT. For the case *η* = 1 and in the limit 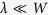, this equation correctly predicts that the transmitted intensity should be proportional to *W*. The beauty of this approach is that the transmission efficiency can be accurately measured in experiment simply by imaging the transmitted surface acoustic field and by means of a reference imaging experiment on an identical sample with no bridge structure.

For the above case of a bridge of length *L* = 7 μm, one finds from this analysis that at *f* = 359 MHz (Fig. 2(e)) the transmission efficiency *η* = 5.7 corresponds to EAT, whereas at 251 MHz (see Fig. 2(d)) *η* = 0.5, which does not reach the criterion for EAT. To understand this behaviour in more detail we have plotted the spectrum *η*(*f*) in Fig. 3. In addition to the lowest-frequency peak, other peaks associated with EAT are found at frequencies *f* = 718, 1006, 1365 and 1724 MHz, whereas regions with *η* ≈ 1 exist between them. Their associated transmission efficiencies are, respectively, *η* = 1.8, 2.7, 3.2 and 3.4. The frequency *f* = 359 MHz corresponds to the first vibrational mode of the bridge for the *x*-direction (in which all points move in phase) with nodes of out-of-plane particle velocity near the bridge ends, as shown by the form of the acoustic field in Fig. 2(e) (both at the surface and in section) and by the line-plot snapshot of the out-of-plane particle velocity plotted along the bridge-length (*x*) direction shown just below the bridge in the figure. Similarly, the four successive peaks in the insets (a–d) of Fig. 3 correspond to the second, third, fourth and fifth modes for the *x*-direction corresponding to Fabry-Perot resonances of the bridge, as is clear from the respective line-plot snapshots.

The resonant frequency of a straight acoustic waveguide can be estimated and compared to the EAT frequencies. Vibrational resonances of the narrow (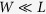) subwavelength bridge are analogous to those of an open air-filled tube. The resonant frequencies can thus be estimated as^31^


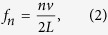


where *n* is the mode order (i.e. the number of half wavelengths along the bridge length) and *v* is the relevant surface acoustic wave velocity (*v* = 4900 ms^−1^ in our case^26^,^32^). Owing to the small bridge width 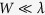 for the frequency range of interest, modes corresponding to resonances in the *y* direction are precluded. Using Eq. (2) with length *L* = 7 μm, the predicted resonances are at *f_n_* = 350, 700, 1050, 1400 and 1750 MHz for *n* = 1–5, corresponding to resonant effective lengths of *λ*/2, *λ*, 3*λ*/2, 2*λ* and 5*λ*/2, respectively, where *λ* is the acoustic wavelength. The agreement with the simulated peaks in transmission efficiency is very good (to within 2.0% overall for *n* = 1–5). The inclusion of an end correction, by analogy to organ-pipe physics, leads to worse fits, and so it was not implemented^31^. For the *n* = 1 resonance, the bridge width (*W* = 0.25 μm) corresponds to *λ*/58; for *n* = 5 it corresponds to ~*λ*/11. We are therefore clearly in the regime of a deeply-subwavelength-bridge aperture required for EAT to occur.

**Figure 4:**
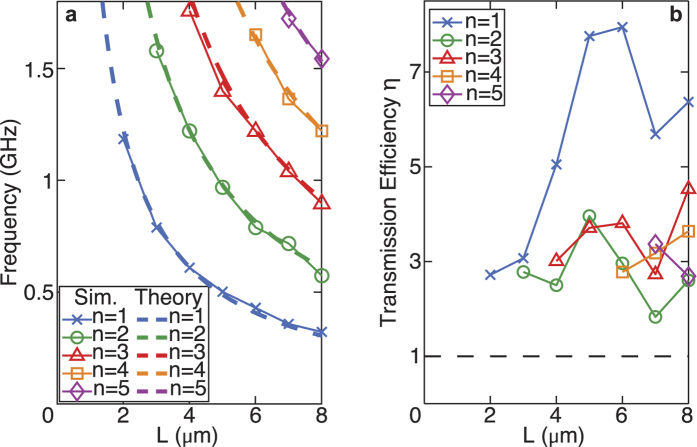
Simulated and predicted EAT frequencies, and simulated transmission efficiencies, for different deeply-subwavelength bridge lengths. (**a**) Simulated EAT peak frequencies associated with the first five modes of a straight bridge as a function of the bridge length *L* (full lines), and compared to the analytical formula of Eq. (2) (dashed lines). Sim.: simulation. (**b**) Plot of the transmission efficiency *η* versus *L*. The condition *η* = 1 is indicated by a dashed line.

Simulations were carried out to investigate the effect of varying the bridge length *L* for 2 ≤ *L *≤ 8 μm in steps of 1 μm. For each case, *η*(*f*) is calculated and the EAT peaks identified, as summarized in Fig. 4(a) together with the predictions of Eq. (2). The agreement is again very good, the mean discrepancy over the 23 detected resonances being ~2.5%. This demonstrates that one can tune the EAT frequency using the bridge length *L* as a variable.

Figure 4(b) shows *η*(*f*) versus *L*, indicating the occurrence of EAT for all cases. The first-mode (*n* = 1) transmission efficiency is consistently larger than that for the four higher-order modes, which show little *L* dependence and *η* ~ 2–3. In contrast, for the first mode *η* rises rapidly with *L* up to *L* = 5 μm, where a maximum *η* ≈ 8.0 is observed. These values are of a similar order to the transmission efficiencies for EAT observed for condensed-matter systems (e.g., *η* = 2.9 in ref. 10 and *η* = 8.3 in ref. 11), but somewhat smaller than values reported for systems based on zero-mass metamaterials in air-filled tubes (e.g., *η* = 57 in ref. 16). The higher value of *η* for *n* = 1 compared to the other modes correlates with the smaller bandwidth of the resonance observed in Fig. 3 (although the Q factor, ~10, is smaller than for the higher-order modes). For *L *> 6 μm and *n* = 1, *η* shows a general downward trend.

### Bridges containing a resonant cavity

By analogy with the use of Helmholtz resonators in air^9^,^18^, we consider the possibility of adding a resonant cavity to the straight bridge. A region of cross section *d* × (2*r* + *W*) is added to the centre of the subwavelength bridge (see Fig. 1(b)), leaving two straight bridge sections of horizontal cross section 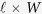 on both sides of this ‘cavity’, where 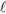 = 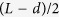.

**Figure 5:**
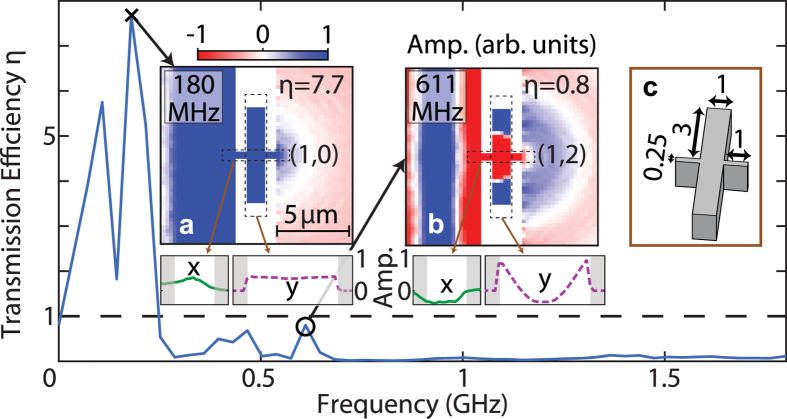
Simulated transmission efficiency spectrum and acoustic fields for a straight bridge containing a rectangular cavity which is relatively thin in the *x* direction. Spectrum of the transmission efficiency *η*(*f*) for a straight bridge containing a cavity, characterized by dimensions *r* = 3, *d* = 1, and 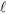 = 1 μm. The condition *η* = 1 is indicated by the dashed lines. Insets (**a**,**b**): acoustic fields for frequencies corresponding to the peaks in *η* between 0.18 and 1.8 GHz, including line-plot snapshots of the average out-of-plane particle velocity plotted along directions *x* and *y*. Inset (**c**): schematic diagram of the top part of the bridge and cavity. Mode identification (*n*, *m*) is also included. Dimensions are in microns. The colour scale of the image plots is the same as in Fig. 3.

We first present a simulation corresponding to a structure with 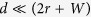, i.e. *r* = 3, *d* = 1 and 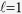 μm, as shown in inset (c) of Fig. 5. The spectrum *η*(*f*) is shown in Fig. 5. In this example, where a relatively thin (*d* = 1 μm) cavity is chosen, we resolve two main peaks in the spectrum. The acoustic field displacement at 108 MHz does not seem to correspond to any recognizable mode, and it remains at present unclear why it occurs. By inspection of the acoustic field line-snapshots in both the *x* and *y* directions in the inset (a) of Fig. 5, it is, however, clear that the next peak at 180 MHz corresponds to EAT with mode number *n* = 1 for the *x* direction, with a transmission efficiency *η* = 7.7. The next well-defined but smaller peak at 611 MHz exhibits a much weaker value of *η* ~ 0.8, which does not correspond to EAT, and inspection of the corresponding acoustic field line-snapshots (inset (b) of Fig. 5) shows that this corresponds to a two-dimensional mode involving resonances in both the *x* and *y* directions: this can be classified by the mode number set (*n*, *m*) = (1, 2), where *n*, the mode number (as used previously for the bridge alone), is the number of half wavelengths in the *x* direction and *m*, the mode number for the *y* direction, is the number of half wavelengths in the *y* direction in the cavity part of the structure. For this lateral resonance, in contrast to the case of the bridge alone, antinodes of the out-of-plane particle velocity exist near the cavity lateral extremities in contact with air, with a corresponding resonant effective length of *λ* in the *y* direction. (The mode associated with the peak around 467 MHz (*η* = 0.7) is not clearly identifiable from its acoustic field, and is not observed for other studied cases of this type of resonator). Inspection of the acoustic field in the frequency region between modes (1, 0) and (1, 2) does not reveal the (1, 1) mode. This is not surprising if one considers that because of the structure’s symmetry the (1, 1) mode should exhibit a node at the midpoint of the cavity, and therefore cannot be excited from this point, i.e. precisely the point where surface acoustic waves are incident on the cavity from the connecting (bridge) section. The *n* = 1 mode at 180 MHz can be classified under this scheme as (1, 0), as there is no spatial variation in the amplitude in the *y* direction.

Simulations were subsequently performed for variations on this example with different values of the lateral cavity dimension *r* (keeping fixed values of 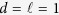 μm) over the range 1 ≤ *r *≤ 8 μm (see Supplementary Information for full details). In all cases, only modes with even *m* are observed, as expected, namely modes (1, 0), (1, 2) and (1, 4), the (1, 4) mode being observed for *r* ≥ 6 μm. The mode (1, 3) is not observed for the same reasons of symmetry as for the mode (1, 1). This latter mode consistently produces strong EAT, with a maximum of 10.5 for *r* = 2 μm over the range of *r* studied, whereas higher modes do not exhibit EAT (except for the case *r* = 1 μm, for which *η* = 1.8 for mode (1, 2)).

The fundamental difference here compared to the straight-bridge case is the occurrence of resonances in both the *x*- and *y*-directions inside the cavity itself. A simple analytical approach to understanding the resonant frequencies for the case 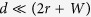 would be by the use of Eq. (2). However, the vibrational coupling between the cavity and bridge sections renders this approach inaccurate. For example, the resonance frequency 611 MHz of mode (1, 2) for the case of the structure of Fig. 5(c) does not closely match that expected for a cavity of length 2*r* + *W* = 6.25 μm (*f*_2_ = 784 MHz). Moreover, the resonance frequency 180 MHz of mode (1, 0) does not match that expected from Eq. (2) for a bridge of length *L* = 3 μm (*f*_1_ = 817 MHz). Presumably the greater mass of the resonant structure plays a role in reducing the observed values compared to these simply estimated resonant frequencies.

**Figure 6:**
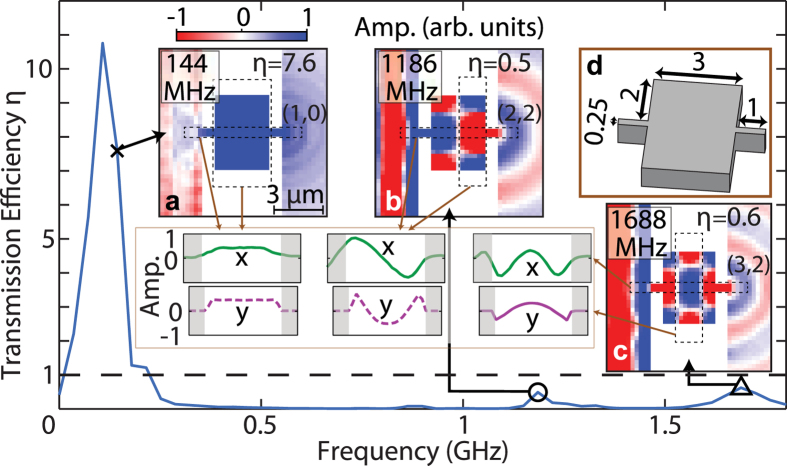
Simulated transmission efficiency spectrum and acoustic fields for a straight bridge containing a cavity with similar dimensions in the *x* and *y* directions. Spectrum of the transmission efficiency *η*(*f*) for a straight bridge containing a cavity, characterized by dimensions *r* = 2, *d* = 3, and 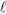 = 1 μm. The condition *η* = 1 is indicated by the dashed lines. Insets (**a**–**c**): acoustic fields for frequencies corresponding to the peaks in *η* between between 0.14 and 1.8 GHz, including line-plot snapshots of the average out-of-plane particle velocity plotted along directions *x* and *y*. Inset (**d**): schematic diagram of the top part of the bridge and cavity. Mode identification (*n*, *m*) is also included. Dimensions are in microns. The colour scale of the image plots is the same as in Fig. 3.

To better understand the cavity resonances, we now consider the example *r* = 2, *d* = 3 and 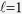 μm shown in inset (d) of Fig. 6, corresponding to a relatively wide cavity. We resolve three recognizable peaks in the spectrum of *η*(*f*), as shown in Fig. 6. Inspection of the acoustic field and the *x* and *y* line-snapshots for the lowest-frequency mode at 144 MHz (inset (a) of Fig. 6) shows that this is mode (1, 0), and corresponds to EAT with *η* ≈ 7.6. The acoustic field at the peak frequency 108 MHz is not associated with a resonant mode of the cavity, and, as noted in the context of Fig. 5 for the same frequency, its origin is unclear. As mentioned in the context of Fig. 5, the observed frequency of mode (1, 0) is significantly smaller than the prediction *f*_1_ =  490 MHz of Eq. (2) for a bridge of length *L* = 5 μm. The two other main peaks in the spectrum occur at 1.186 and 1.688 GHz, and correspond to modes (2, 2) and (3, 2) (insets (b) and (c)) with *η* ~ 0.5 and ~0.6, respectively. One can also discern another very small peak at 862 MHz corresponding to mode (1, 2) with *η* ~ 0.1.

There is an apparent similarity of the simultaneous *x*- and *y*-direction resonances for modes (2, 2) and (3, 2) with membrane resonances, i.e. for dispersionless waves confined in a rectangular area^33^:


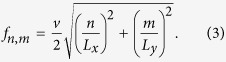


We can set, as an approximation, *L*_*x*_ = *d* and *L*_*y*_ = 2*r* + *W* as the dimensions of the cavity in the *x* and *y* directions (with boundary conditions for the *x* direction assumed to be the same as those in the *y* direction, i.e. an antinode in velocity at the cavity edges). The frequencies of modes (2, 2) and (3, 2) would, according to a simplistic use of this equation with *v* = 4900 ms^−1^ as before, be expected at 2.00 and 2.71 GHz, values which are not in good agreement with the respective simulated values 1.186 and 1.688 GHz. However, simulations for the cavity part of the structure in isolation (see Supplementary Information), show that without the bridge sections one should use mode number *n* − 1 in place of *n* in Eq. (3) for *n* ≥ 1 to produce the correct mode shape in the cavity section. Using this modification, the analytically predicted frequencies for modes (2, 2) and (3, 2) shown in Fig. 6(b,c), respectively, become 1413 and 1999 MHz, in closer agreement with the respective simulated values 1186 and 1688 MHz. For the mode (1, 2), Eq. (3) with *n* reduced by 1 to give 0 is equivalent to Eq. (2). The predicted frequency (784 MHz) only matches the simulated resonance frequency (611 MHz) approximately, but the prediction is much better than that (2.57 GHz) obtained with the unmodified form of Eq. (3). Use of Eq. (3) in its modified form is therefore reasonably good to an accuracy of better than ~30% for the cases considered. (This error applies for all the geometries treated in the Supplementary Information, except for the ones with 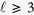 μm for which the resonant frequencies cannot be properly compared with Eq. (3) because of the strong influence of the connecting bridge sections).

**Figure 7:**
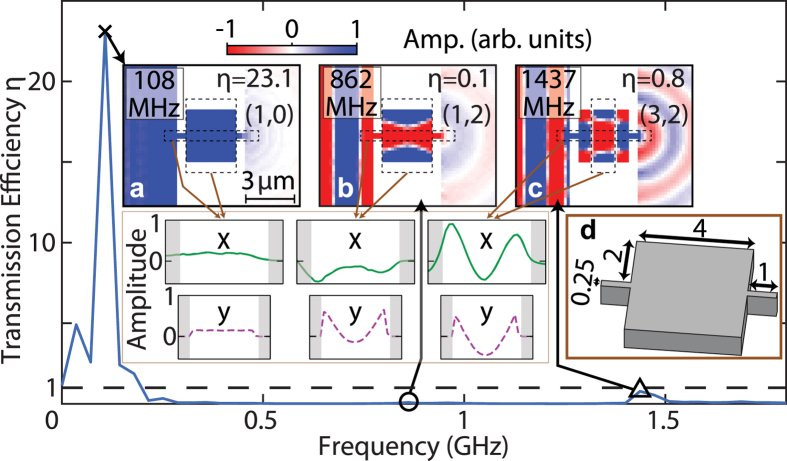
Simulated transmission efficiency spectrum and acoustic fields for a straight bridge containing a cavity with similar dimensions in the *x* and *y* directions. Spectrum of the transmission efficiency *η*(*f*) for a straight bridge containing a cavity, characterized by dimensions *r* = 2, *d* = 4, and 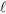 = 1 μm. The condition *η* = 1 is indicated by the dashed lines. Insets (**a**–**c**): acoustic fields for frequencies corresponding to the EAT peaks in *η* between 0.1 and 1.8 GHz, including line-plot snapshots of the average out-of-plane particle velocity plotted along directions *x* and *y*. Inset (**d**): schematic diagram of the top part of the bridge and cavity. Mode identification (*n*, *m*) is also included. Dimensions are in microns. The colour scale of the image plots is the same as in Fig. 3.

Finally, we consider the example *r* = 2, *d* = 4 and 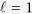 μm, shown in inset (d) of Fig. 7, corresponding to a cavity that is 1 μm longer compared to the previous example in Fig. 6. The mode (1, 0) is clearly visible on inspection of the acoustic field and *x* and *y* line-snapshots for the lowest frequency mode at 108 MHz (inset (a) of Fig. 7), showing EAT with *η* 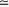 23 that corresponds to the highest transmission efficiency found among the different geometries studied (see Supplementary Information). For this case, inspection of the acoustic field at 108 MHz shows that this mode corresponds to a bone fide resonant mode of the cavity. The increase of 1 μm in *d* compared to the example of Fig. 6 is enough to change the enhancement factor of mode (1, 0) from 7.6 to 23.1. This demonstrates that small modifications in geometry can lead to very different transmission efficiencies. Modes (1, 2) and (3, 2) are also clearly visible at higher frequencies in Fig. 7, but with transmission efficiencies of only 0.1 and 0.8, respectively. Mode (2, 2), clearly detected for *d* = 3 μm, is no longer resolvable. As was the case for the data of Fig. 6, the position of the resonance peaks for the data of Fig. 7 are also in better accord with the predictions of Eq. (3) with *n* replaced by *n* − 1.

It is clear that the addition of the cavity to the bridge structure makes the analytical prediction of the resonant frequencies less accurate. For both the cases of the bridge alone and the bridge containing a resonant cavity, the transmission efficiency *η* is not simply determined. In particular, it appears that the cavity often acts to quench the EAT corresponding to higher-order-resonances, although it is not yet clear in general which resonant modes should exhibit EAT and which should not. It is, however, clear that the transmission efficiency depends on the geometry and on acoustic losses (see Methods). Our results demonstrate that a deeply subwavelength bridge without an associated cavity can show *η* ~ 8 for the first resonant mode, and that higher-order modes can also exhibit significant EAT (up to *η* ~ 5). When adding a resonant cavity, the transmission efficiency for the first resonant mode (1, 0) can be enhanced (up to 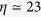), whereas corresponding higher-order modes do not exhibit *η* higher than 2 (see Supplementary Information). It is not entirely clear why the addition of a cavity is an advantage for producing high EAT efficiencies. One can speculate that a higher Q factor (not easily determined in our study because of the finite frequency resolution) for the lateral resonance may be responsible. In this connection it may be significant that without the cavity the transmission efficiency at off-resonance frequencies remains around ~1 whereas in the presence of the cavity it remains ~0.

## Discussion

In conclusion we have demonstrated the phenomenon of extraordinary transmission of surface waves. We achieve this by means of acoustic simulations in crystalline Si for two different types of structure, a straight bridge of deeply subwavelength width and one containing a rectangular cross-section cavity. We show how different surface vibrational modes can give rise to EAT, provided that mode excitation is not precluded by symmetry considerations. In the case of a straight bridge, the mode frequencies are very well accounted for through the Fabry-Perot resonances of surface acoustic waves on the bridge. For a bridge containing a cavity, the presence of the bridge sections complicates the situation, preventing accurate analytical prediction of the resonant frequencies.

The transmission efficiency *η* was also estimated from the observed surface acoustic fields. In the case of both types of structure, EAT can be observed; we find transmission efficiencies up to *η* ~ 23. We also investigated how *η* varies with the structure geometry. In the case of the straight bridge, the *n* = 1 mode, with the longest wavelength, consistently gave the highest value of *η*. For the bridge containing a cavity, an equivalent mode, labelled (1, 0), also gives rise to the highest value of *η*, whereas the modes involving lateral resonances (labelled by (*n*, *m*) with *m* ≥ 1) give values of *η* up to ~2.

Understanding the detailed loss processes for each type of resonance is required in order to clarify the upper limit for the transmission efficiency. We have only studied two types of cavity, and many more possibilities exist. Losses occur by transmission to the bulk, and so extending this work to Lamb waves in plates would also very probably be beneficial for obtaining higher values of *η*. The present work shows that reducing the dimensionality of the extraordinary transmission problem to waves confined to a plane does not prevent the phenomenon from existing. Our work therefore opens the way to similar investigations on extraordinary transmission of other forms of surface waves, including water waves and surface plasmon polaritons. This study should therefore stimulate experimental work on extraordinary transmission of surface waves not only in acoustics but also in these other fields, and lead to new perspectives in surface-wave metamaterials and subwavelength imaging.

## Methods

The simulations are conducted with a commercial time-domain finite-element method (FEM) package PZFlex (Weidlinger A. Inc.). The *x* and *y* directions (see Fig. 1) correspond to the [0 1 1] and [0 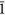 1] crystal axis orientations, respectively. The silicon is modelled with the following mechanical stiffness constants^32^: *c*_11_ = 166, *c*_44_ = 79.6 and *c*_12_ = 63.9 GPa. The three-dimensional elements, each consisting of eight nodes arranged on an orthogonal grid, have a volume corresponding to 0.125 × 0.125 × 0.125 μm^3^, leading to a total of ~3 × 10^6^ elements. Although this chosen size leads to only two elements over the bridge width (*W*), simulations carried out with elements of 250 and 62.5 nm^3^ (i.e., with 1 and 4 elements on the bridge thickness) show that similar results (to within ~10% accuracy) are obtained for these two cases. We therefore choose 125 nm^3^ as the element volume, giving a good compromise between the computation time and the calculation accuracy. The simulation duration is 27.6 ns, with temporal step of 10.6 ps.

As we are dealing with surface acoustic waves, it is well known that a non-negligible part of the acoustic amplitude can propagate in the depth (*z*-axis) direction when reaching a 90° corner; an estimate of the amplitude reflection and transmission coefficients at such a corner^34^ can be made by approximating crystalline Si to an isotropic solid^35^ with Poisson’s ratio 0.22, yielding 0.3 and 0.68 for the respective coefficients. In practice, it is clear from the Q factors obtained for the transmission resonances that, for 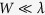, an enhanced reflection coefficient compared to this estimate is expected. This is not surprising in the cases *W* or 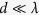, as exemplified in the case of organ pipes^36^, owing to the increase in acoustic impedance mismatch under these conditions. Moreover, simulations (see Supplementary Information) show that surface waves can be confined at the end of a rectangular-cross-section rod as well-defined vibrational modes.

## Additional Information

**How to cite this article**: Mezil, S. *et al*. Extraordinary transmission of gigahertz surface acoustic waves. *Sci. Rep.* **6**, 33380; doi: 10.1038/srep33380 (2016).

## Supplementary Material

Supplementary Information
