# Supplementary material for: Extraordinary transmission of gigahertz surface acoustic waves
Source: Sci Rep. 2016 Sep 19;6:33380. doi: 10.1038/srep33380 (PMC5027576; doi:10.1038/srep33380)
Supplement: Supplementary Information [file srep33380-s1.pdf]

SUPPLEMENTARY INFORMATION

Extraordinary transmission of gigahertz surface acoustic waves

Sylvain Mezil,[1] Kazuki Chonan,[1] Paul H. Otsuka,[1] Motonobu Tomoda,[1] Osamu Matsuda,[1] Sam H. Lee,[2] and Oliver B. Wright[1, a)]

[1)] *Division of Applied Physics, Graduate School of Engineering, Hokkaido University, Sapporo 060-8628, Japan*

[2)] *Institute of Physics and Applied Physics, Yonsei University, Seoul 120-749, Korea*

This Supplementary Information covers the results of the simulations of resonant frequency and transmission efficiency for surface acoustic waves in crystalline silicon when varying the dimensions of a subwavelength bridge containing a cavity. We also present simulations of the vibrational modes of a surface-wave cavity, and compare with the simulated extraordinary-acoustic-transmission frequencies and analytically-calculated frequencies.

[a)] olly@eng.hokudai.ac.jp

I. VARIATIONS IN THE CAVITY DIMENSIONS

Simulations were carried out to better understand the extraordinary-acoustic transmission (EAT) phenomenon with a cavity characterized by the dimensions r, d and ℓ, as shown in Fig. 1 of the main text. The straight bridge sections have a horizontal cross section $\ell \times W$ on both sides of the cavity of dimensions $d \times (2r + W)$. We varied the parameter r from 1 to 8 µm keeping ℓ and d constant at 1 µm (Fig. S1(a)), the parameter d from 1 to 8 µm with $\ell = 1$ and $r = 2$ µm (Fig. S1(b)), and finally the dimension ℓ from 0.5 to 5 µm with $d = 1$ and $r = 2$ µm (Fig. S1(c)).

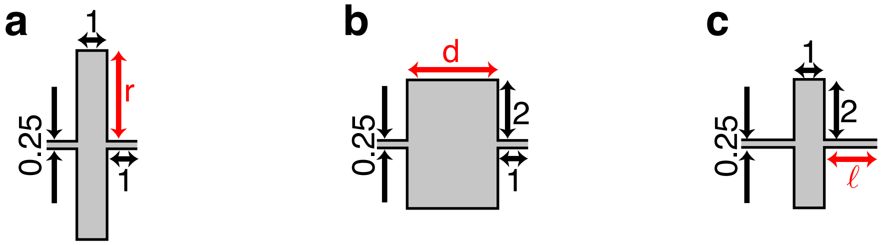

FIG. S1. **Top view of the bridge and cavity resonator geometries** for the considered simulations varying (a) the length r with $\ell = d = 1$ µm, (b) the length d with $\ell = 1$ and $r = 2$ µm, and (c) the length ℓ with $d = 1$ and $r = 2$ µm.

A. Influence of the length r

The evolution of the frequency and the transmission efficiency of the bridge and cavity modes as a function of the length r are shown in Fig. S2(a) and (b), respectively, with r in the range 1 to 8 µm (see Fig. S1(a)). Three modes are detected: $(1, 0)$, $(1, 2)$ and $(1, 4)$.

The first mode, corresponding to mode $(1, 0)$, is not very sensitive to the variation of r. (The frequency evolves from 359 to 108 MHz when r varies from 1 to 8 µm.) Without the cavity ($r = 0$), the frequency of mode $(1, 0)$ is found to be 862 MHz, showing that the addition of the cavity, regardless of its size, has a significant influence on the mode frequency. The transmission efficiency η for this mode exhibits and irregular variation with frequency, showing two local maxima at $r = 2$ and 7 µm. For these values of r, we detect a transmission efficiency of $\eta = 10.5$ and 10.8, respectively.

The frequency of mode $(1, 2)$ is very sensitive to the value of r, and exhibits an evolution

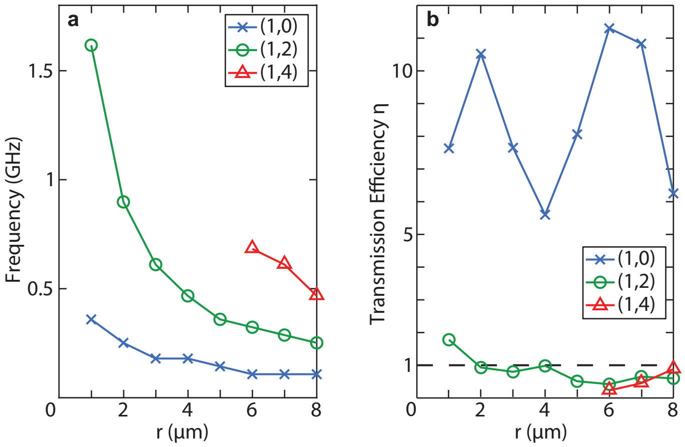

FIG. S2. (a) **Simulated peak frequencies associated with the modes of a bridge containing a cavity as a function of the length r with fixed parameters $\ell = 1$, $d = 1$ µm.** (b) Plot of the corresponding transmission efficiency η versus r. The condition $\eta = 1$ is indicated by the dashed line.

$\propto 1/r$ similar to the behaviour found for the straight bridge ($f_n \propto 1/L$ in Eq. (2)). The transmission efficiency shows an overall decrease as r increases. EAT is observed only for $r = 1$ ($\eta = 1.8$). Although this mode is still detected for higher values of r, it does not produce EAT (the transmission efficiency varies between 0.5 and 0.98 for $r \geq 2$).

Finally, the mode $(1, 4)$ is only detected for relatively large values of $r \geq 6$ µm. It is clear from the resonant frequencies involved ($f \sim 500$ MHz) that it would also be expected to appear for the lower values of r investigated at frequencies up to 1.8 GHz. Its absence in this higher frequency range is attributed to η becoming too low to be resolved. In the range of r from 6 to 8 µm, one can observe a rise in η with frequency for this mode $(1, 4)$.

B. Influence of the length d

The evolution of the frequency and the transmission efficiency of the bridge and cavity modes as a function of the length d are shown in Fig. S3(a) and (b), respectively, with d in the range 1 to 8 µm (see Fig. S1(b)). Seven modes are detected. The frequencies of modes $(1, 0)$ and $(1, 2)$ are not very sensitive to the value of d, whereas those for the five other modes show a stronger dependence $\propto 1/d$.

Only the lowest mode (1,0) exhibits EAT for all these cases, and displays a local maximum

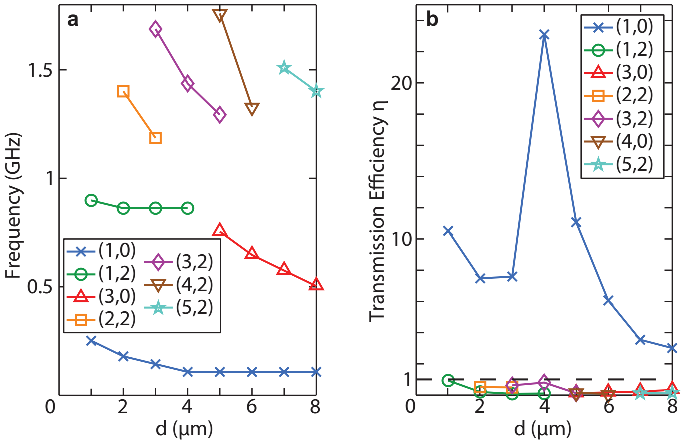

FIG. S3. (a) **Simulated peak frequencies associated with the modes of a bridge containing a cavity as a function of the length d with fixed parameters $\ell = 1$, $r = 2$ μm.** (b) Plot of the corresponding transmission efficiency η versus d. The condition $\eta = 1$ is indicated by the dashed line.

when $d = 4$ μm (see Fig. 7 in the main text). The other six detected modes do not display EAT for all the variations of d studied, exhibiting transmission efficiencies between ~ 0.1 and 0.9. The origin of the local maximum at $d = 4$ μm is unknown, and merits further study.

C. Influence of the length ℓ

The evolution of the frequency and the transmission efficiency of the bridge and cavity modes as a function of the length ℓ are shown in Fig. S4(a) and (b), respectively, with ℓ in the range 0.5 to 5 μm (see Fig. S1(c)).

A non-identified mode, denoted NI, is detected for ℓ = 4-5 μm. Its frequencies seem to lie on the extrapolation of the those for mode $(1, 2)$, but the particle-velocity fields (not reproduced here) do not match those of $(1, 2)$. The acoustic fields of these NI modes indicate that the associated cavity motion is in antiphase to those of the bridge sections on either side. The particle-velocity profile along the y-axis in the cavity corresponds to mode m=1. Along the x-axis, each part of the bridge contains half of a period (with a minima at each extremity) in antiphase with the cavity variation. This would naturally correspond to mode m=3 if the length of the cavity was comparable to the length of each bridge section, which

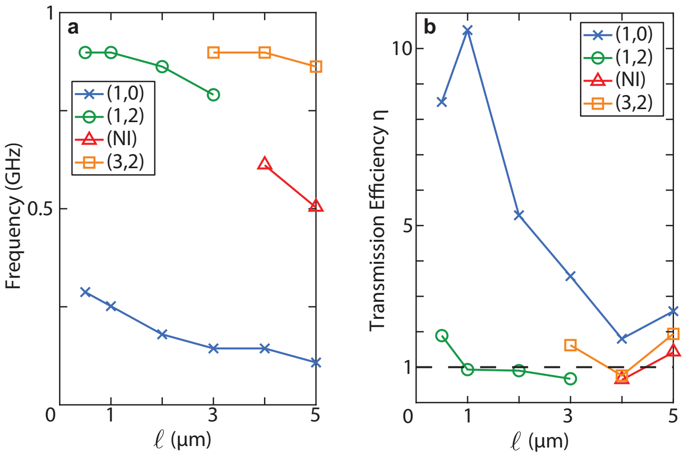

FIG. S4. (a) **Simulated peak frequencies associated with the modes of a bridge containing a cavity as a function of the length ℓ with fixed parameters $d = 1$, $r = 2$ µm.** (b) Plot of the corresponding transmission efficiency η versus ℓ. The condition $\eta = 1$ is indicated by the dashed line. NI: Non-identified mode

is not the case ($\ell = 4$ or 5 µm, whereas d=1 µm).

The behaviour of both the peak frequency and transmission efficiency for all the detected modes are quite different from the previous cases for which r or d were varied. The peak frequencies do not exhibit a $1/\ell$ variation; instead they are only weakly dependent on ℓ. The transmission efficiency of the first mode $(1, 0)$ is much lower than for the previous cases for $\ell \geq 2$ µm. Unlike the above two cases for which r and d were varied, the transmission efficiencies of the higher-order modes are mostly above η=1, with a minimum of $\eta = 0.7$ for mode (1,2) at $\ell = 3$ µm and a maximum of $\eta = 1.9$ for modes (1,0) and (3,2) at $\ell = 0.5$ and 5 µm, respectively.

II. VIBRATIONAL MODES OF A RECTANGULAR SURFACE-WAVE CAVITY

Surface-wave cavities bounded by free space do not appear to have been studied, so in order to obtain a better understanding of the surface-wave modes in such cavities, we have carried out simulations of the vibrational modes a cavity formed at one end of an isolated rectangular bar of Si. These frequency-domain simulations are conducted with a commercial finite-element method (FEM) package (COMSOL Multiphysics 5.1) on typical geometries

corresponding to those of Figs. 5 and 6 in the main text. Symmetric and antisymmetric boundary conditions are used to reduce the required simulation volume by a factor of 4, so the Si volumes studied are $0.5\times3.125\times70$ μm^3 for the geometry corresponding to Fig. 5 of the main text and $1.5\times2.125\times70$ μm^3 for that of Fig. 6. Absorbing domains (known as infinite-element domains in COMSOL) of 5 μm thickness are used to represent semi-infinite rods. As with the calculations involving PZFlex, the x and y directions correspond to the [0 1 1] and [0 $\bar{1}$ 1] Si crystal axis directions, respectively, and we make use of the same values of density and elastic constants. The tetrahedral element size is selected to be less than 0.5 μm (less than 1/5 of the surface-wave wavelength at 2 GHz), leading to a total of 1.2×10^4 elements for the cavity based on Fig. 5 of the main text, and 1.5×10^3 elements for the cavities based on Fig. 6. The COMSOL eigenfrequency solver calculates numerous eigenmodes for frequencies up to 2 GHz, but most of them have the elastic energy distributed over the whole bar. By selecting the eigenmodes whose elastic energy distribution is concentrated in the near-bar-end region with the required spatial symmetry (e.g., excluding torsional modes that are not excited in our case), the vibrational eigenmodes of interest can be extracted.

Figure S5 shows the simulated mode patterns at the end of an isolated rectangular cross-section bar of Si for three representative cases. For a cavity with $2r + W = 6.25$ and $d = 1$ μm, the mode $(0, 2)$ appears at 595 MHz (see Fig. S5(a)), similar to the frequency 611 MHz found for mode $(1, 2)$ in inset (b) of Fig. 5 in the main text. The closeness of these frequencies may be thanks to the relatively short bridge sections used for the EAT structure. The form of the mode $(1, 2)$ at 611 Hz is also very similar, as is evident by comparison of the x and y line snapshots of the mode shapes in the inset (b) of Fig. 5 of the main text and in Fig. S5(a). Analytic theory based on Eq. (3) for mode $(0, 2)$ gives a frequency of 784 Hz. These results are summarized in Table I. The simulated frequency for the isolated cavity is therefore somewhat closer to that of the EAT structure than the analytical prediction, showing, not surprisingly, that the analytical prediction is somewhat oversimplified. The different values of the x mode numbers, i.e. n=1 for the EAT structure and $n-1$=0 for the isolated cavity, arise because of the difference in boundary conditions: for the EAT structure there are nodes in out-of-plane surface particle velocity at the edges (for the x direction) of the vibrating region (i.e. including the bridge sections), whereas for the isolated cavity there are antinodes.

For the cavity with $2r + W = 4.25$ μm and $d = 3$ μm, the modes $(1, 2)$ and $(2, 2)$ appear

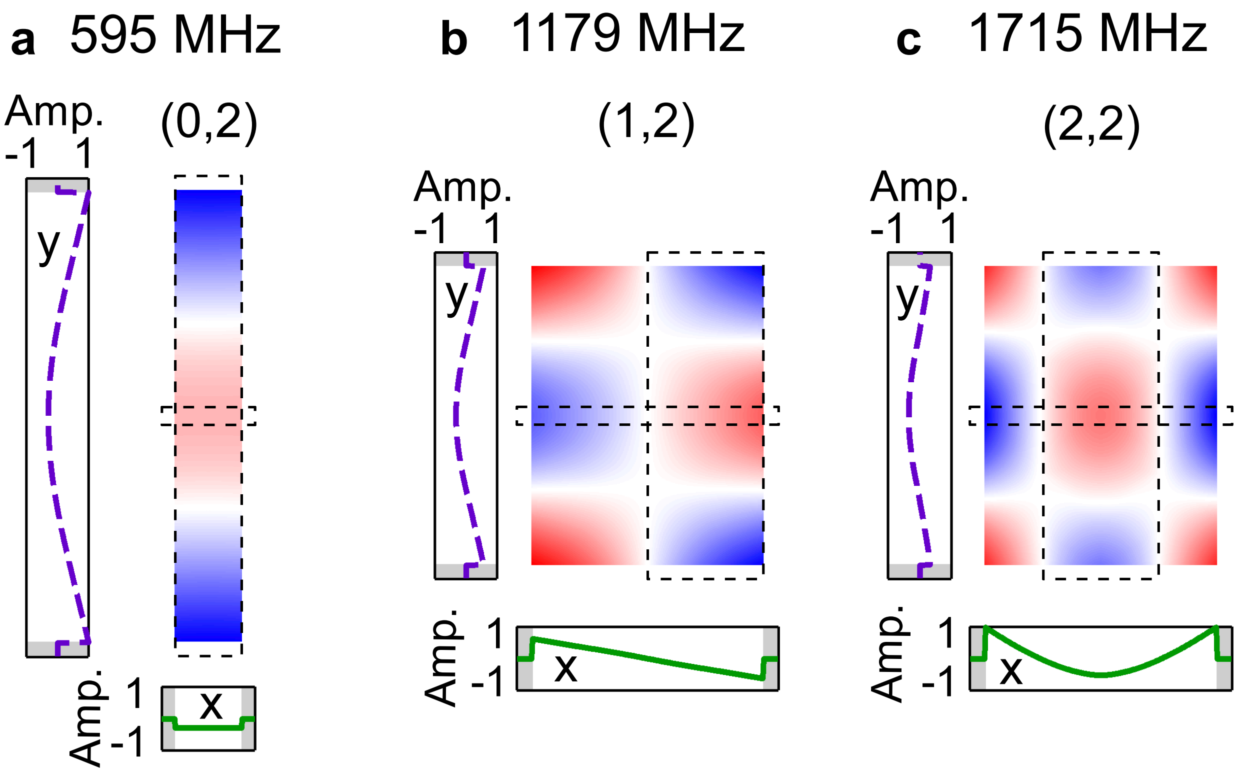

FIG. S5. **Simulated mode patterns for the out-of-plane particle velocity for vibrations localized in an effective cavity at the end of a bar of Si of rectangular cross section.** (a) The case of a cavity with $2r + W = 6.25$ and $d = 1$ µm for mode $(0, 2)$, closely related to the mode $(1, 2)$ of Fig. 5(b) of the main text. (b) The case of a cavity with $2r + W = 4.25$ and $d = 3$ µm for mode $(1, 2)$, closely related to the mode $(2, 2)$ of Fig. 6(b). (c) The case of a cavity with $2r + W = 4.25$ and $d = 3$ µm for mode $(2, 2)$, closely related to the mode $(3, 2)$ of Fig. 6(c). x and y line-plot snapshots of the fields are also shown. The Si crystal orientation is the same as the cavity of the main text.

at 1179 and 1715 MHz (see Fig. S5(b) and (c)). These are very similar in form to the modes $(2, 2)$ at 1186 MHz and $(3, 2)$ at 1688 MHz noted in insets (b) and (c), respectively, of Fig. 6 in the main text, as is evident by comparison of the x and y line snapshots of the mode shapes in these insets and those in Fig. S5(b) and (c). Analytic theory based on Eq. (3) for modes $(1, 2)$ and $(2, 2)$ give frequencies 1425 and 2242 MHz, respectively, values which are not so different from simulation. However, as with the case of the cavity of Fig. 5, the simulated frequencies for the isolated cavities are somewhat closer to those of the EAT

structure than those obtained by analytical predictions. The frequencies for this second cavity geometry are also summarized in Table I.

TABLE I. **Table to compare resonances frequencies calculated for rectangular Si surface-wave cavities in three different ways.** This is done by means of 1) simulations (sim.) based on EAT for a cavity containing a bridge structure, 2) simulations based on the vibrational modes of the end of an isolated rectangular cross-section bar, and 3) analytical theory based on Eqs. (2) or (3) of the main text with v=4900 ms^{-1}. The cavities considered correspond to Figs. 5 and 6 of the main text. For the case of the cavity of Fig. 5 of the main text, the mode numbers (n,m) for the cavity with the bridge connections are interpreted to be $(1, 2)$ (see the x cross section in Fig. 5(b)), whereas for the isolated cavity they are evidently $(0, 2)$ (see the x cross section in Fig. S5(a)). For the case of the cavities of Fig. 6 of the main text, the mode numbers for the cavity with the bridge connections are interpreted to be $(2, 2)$ and $(3, 2)$ (see the x cross section in Fig. 6(b), (c)), whereas for the isolated cavity the corresponding mode numbers are evidently $(1, 2)$ and $(2, 2)$ (see the x cross sections in Fig. S5(b), (c)).

Cavity size (µm× µm)	Cavity+bridge sim. for EAT (MHz)	Isolated cavity sim. (MHz)	Analytic theory (MHz)
1×6.25	$(1, 2)$: 611	$(0, 2)$: 595	$(0, 2)$: 784
3×4.25	$(2, 2)$: 1186	$(1, 2)$: 1179	$(1, 2)$: 1413
3×4.25	$(3, 2)$: 1688	$(2, 2)$: 1715	$(2, 2)$: 1999
